# Hydrogen-bonding patterns in pyrimethaminium pyridine-3-sulfonate

**DOI:** 10.1107/S1600536810029119

**Published:** 2010-07-24

**Authors:** Jeyaraman Selvaraj Nirmalram, Packianathan Thomas Muthiah

**Affiliations:** a School of Chemistry, Bharathidasan University, Tiruchirappalli 620 024, Tamilnadu, India

## Abstract

In the asymmetric unit of the title salt [systematic name: 2,4-diamino-5-(4-chloro­phen­yl)-6-ethyl­pyrimidin-1-ium pyri­dine-3-sulfonate], C_12_H_14_N_4_Cl^+^·C_5_H_4_NSO_3_^−^, there are two independent pyrimethaminium cations and two 3-pyridine sulfonate anions. Each sulfonate group inter­acts with the corresponding protonated pyrimidine ring through two N—H⋯O hydrogen bonds, forming a cyclic hydrogen-bonded bimolecular *R*_2_^2^(8) motif. Even though the primary mode of association is the same, the next higher level of supra­molecular architectures are different due to different hydrogen-bonded networks. In one of the independent molecules in the asymmetric unit, the pyrimethamine cation is paired centrosymmetrically through N—H⋯N hydrogen bonds, generating an *R*_2_^2^(8) ring motif. In the other molecule, the pyrimethamine cation does not form any base pairs; instead it forms hydrogen bonds with the 3-pyridine sulfonate anion. The structure is further stabilized by C—H⋯O, C—H⋯N and π–π stacking [centroid–centroid distance = 3.9465 (13) Å] inter­actions.

## Related literature

For background to crystal engineering and supra­molecular chemistry, see: Desiraju (1989 ▶); Lehn (1995 ▶). For structures involving pyrimethamine carboxyl­ates, see: Sethuraman *et al.* (2003 ▶); Stanley *et al.* (2002 ▶). For structures involving sulfonates, see: Hemamalini *et al.* (2005 ▶); Balasubramani *et al.* (2007 ▶); Baskar *et al.* (2003 ▶). For a survey on hydrogen-bonding patterns involving sulfonate salts, see: Haynes *et al.* (2004 ▶). For the crystal structures of pyrimethamine and metoprine, see: Sethuraman & Thomas Muthiah (2002 ▶); De *et al.* (1989 ▶). For modeling studies on DHFR–PMN complexes, see: Sansom *et al.* (1989 ▶). For hydrogen-bond motifs, see: Etter (1990 ▶); Bernstein *et al.* (1995 ▶).
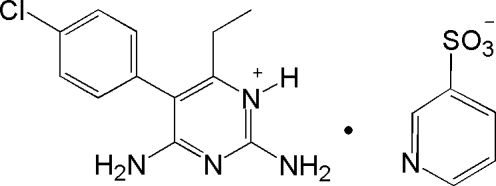


## Experimental

### 

#### Crystal data

C_12_H_14_ClN_4_^+^·C_5_H_4_NO_3_S^−^
*M* *_r_* = 407.88
Triclinic, 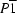

*a* = 10.4525 (17) Å
*b* = 12.200 (2) Å
*c* = 16.539 (3) Å
α = 81.130 (9)°
β = 83.580 (9)°
γ = 68.649 (8)°
*V* = 1937.3 (6) Å^3^
*Z* = 4
Mo *K*α radiation
μ = 0.33 mm^−1^
*T* = 296 K
0.22 × 0.17 × 0.15 mm

#### Data collection

Bruker SMART APEXII CCD area-detector diffractometer
Absorption correction: multi-scan (*SADABS*; Bruker, 2008 ▶) *T*_min_ = 0.930, *T*_max_ = 0.952
38297 measured reflections
11491 independent reflections
7509 reflections with *I* > 2σ(*I*)
*R*_int_ = 0.038

#### Refinement

*R*[*F* ^2^ > 2σ(*F* ^2^)] = 0.048
*wR*(*F* ^2^) = 0.142
*S* = 1.04
11491 reflections
489 parameters
H-atom parameters constrained
Δρ_max_ = 0.36 e Å^−3^
Δρ_min_ = −0.39 e Å^−3^

### 

Data collection: *APEX2* (Bruker, 2008 ▶); cell refinement: *SAINT* (Bruker, 2008 ▶); data reduction: *SAINT*; program(s) used to solve structure: *SHELXL97* (Sheldrick, 2008 ▶); program(s) used to refine structure: *SHELXL97* (Sheldrick, 2008 ▶); molecular graphics: *PLATON* (Spek, 2009 ▶); software used to prepare material for publication: *PLATON*.

## Supplementary Material

Crystal structure: contains datablocks global, I. DOI: 10.1107/S1600536810029119/is2577sup1.cif

Structure factors: contains datablocks I. DOI: 10.1107/S1600536810029119/is2577Isup2.hkl

Additional supplementary materials:  crystallographic information; 3D view; checkCIF report

## Figures and Tables

**Table 1:** Hydrogen-bond geometry (Å, °)

*D*—H⋯*A*	*D*—H	H⋯*A*	*D*⋯*A*	*D*—H⋯*A*
N2*A*—H2*A*2⋯O2*B*^i^	0.86	2.15	2.978 (2)	162
N1*A*—H1*A*⋯O1*A*^ii^	0.86	1.92	2.764 (2)	166
N1*B*—H1*B*⋯O1*B*^iii^	0.86	1.97	2.805 (2)	164
N2*A*—H2*A*1⋯O2*A*^ii^	0.86	2.00	2.808 (2)	157
N4*A*—H4*A*1⋯N3*A*^i^	0.86	2.17	3.027 (2)	178
N4*A*—H4*A*2⋯O2*B*	0.86	2.22	2.909 (2)	137
N2*B*—H2*B*2⋯O3*A*^iii^	0.86	2.15	3.003 (3)	171
N2*B*—H2*B*1⋯O2*B*^iii^	0.86	2.16	3.005 (2)	167
N4*B*—H4*B*1⋯N17*A*^iii^	0.86	2.19	3.033 (3)	168
C10*B*—H10*B*⋯O3*A*	0.93	2.51	3.299 (3)	143
C16*A*—H16*A*⋯N3*B*^iii^	0.93	2.44	3.128 (3)	131
C19*B*—H19*B*⋯O1*A*^iv^	0.93	2.48	3.361 (3)	157

Symmetry codes: (i) 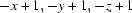; (ii) 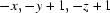; (iii) 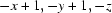; (iv) 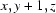.

**Fig. 1.**
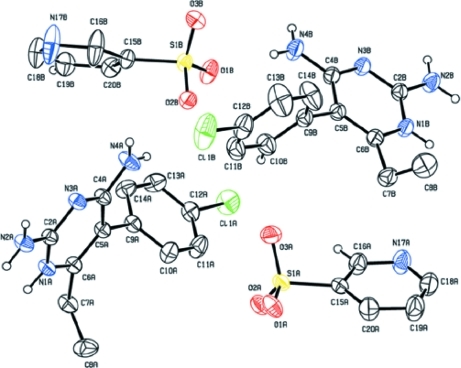
An ORTEP view of the asymmetric unit of the compound, showing the atom-labelling scheme. Displacement ellipsoids are drawn at the 30% probability level. H atoms not involved in the hydrogen-bonds have been omitted for clarity.

**Fig. 2.**
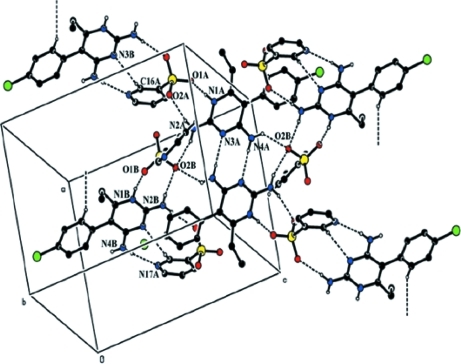
A packing diagram showing the interactions. H atoms not involved in the hydrogen-bonds have been omitted for clarity.

## supplementary crystallographic information

### Comment

A variety of strategies have been adopted by solid state chemist to tailor the physiochemical properties of an active pharmaceutical ingredient (API). One such strategy is to prepare salt forms of these API's using the concept of crystal engineering (Desiraju, 1989) and supramolecular chemistry (Lehn, 1995). We have reported from our laboratory, the crystal structure of pyrimethamine (PMN), (Sethuraman & Muthiah, 2002) an antifolate drug used in antimalarial chemotherapy and treatment of opportunistic infections in patients with AIDS. Investigations of a fairly large number of crystal structures of pyrimethamine salts involving carboxylates (Sethuraman *et al.*, 2003; Stanley *et al.*, 2002) and a few sulfonates (Hemamalini *et al.*, 2005; Balasubramani *et al.*, 2007) have shown an inclination towards the formation of certain robust motifs and a variety of supramolecular architectures. The CSD survey by Haynes *et al.* (2004) on the sulfonate salts, revealed various hydrogen bonding patterns and their preferences with specific functional groups.

It is therefore of interest, to investigate the packing preferences and supramolecular architectures of the title compound (Scheme 1). The compound crystallizes in the triclinic space group *P*1, with two molecules in the asymmetric unit (Figure 1). The crystallographically independent pyrimethamine molecules (A and B) are protonated at N1A and N1B positions as is evident from the increase in respective bond angle at C—N—C, when compared to its neutral form (Sethuraman & Thomas Muthiah, 2002). The bond lengths and angles between the two molecules are in good agreement, with those observed in computer modeling studies on dihydrofolate reductase DHFR–PMN complexes (Sansom *et al.*, 1989) and the crystal structure of metoprine (De *et al.*, 1989).

Each of the protonated pyrimethaminium (N1A and N1B) cations interacts with two oxygen atoms of the respective sulfonate anion through two N—H···O hydrogen bonds, forming an eight membered ring motif *R*_2_^2^(8) (Etter, 1990; Bernstein *et al.*, 1995). It is well known that sulfonates imitate carboxylates in forming such bidentate motifs (Baskar *et al.*, 2003). Despite this analogy, what makes things interesting is the higher level of supramolecular organization assumed by the two independent molecules. The pyrimethaminium cation A is centrosymmetrically paired through N4—H···N3 hydrogen bonds involving the 4-amino group and the N3 atom of the pyrimidine to form the ring motif *R*_2_^2^(8). In addition to the base pairing, one of the sulfonate oxygen atoms (O2B) bridges the 2-amino and the 4-amino groups on both sides. The combination of such base-pairing patterns and the further bridging of the oxygen atom, leads to the formation of a linear array of four hydrogen bonds. The corresponding graph-set notations are *R*_3_^2^(8), *R*_2_^2^(8) and *R*_3_^2^(8). Occurrence of such an array is a characteristic feature observed in structures reported earlier (Stanley *et al.*, 2002).

The pyrimethaminium cation B does not form any base pairs across its inversion related molecule, instead it forms hydrogen bonds with the 3-pyridine sulfonate(A) through N2B—H···O3A, C16A—H···N3B, N4B—H···N17A interactions to form motifs with *R*_2_^2^(9) and *R*_2_^2^(7) graph set notations (Figure 2). Combination of these motifs leads to the formation of a triplet hydrogen bond array. A previous report from our laboratory on a closely related system, pyrimethaminium benzene sulfonate salt did not yield such an array. This might be due to the absence of acceptor in the benzene ring (Balasubramani *et al.*, 2007).

Other than these strong interactions, the crystal structure is stabilized by C—H···O, C—H···N interactions and π–π stacking interactions between the PMN (B) molecules, with a centroid-to-centroid distance of 3.9465 (13) Å, an interplanar spacing of 3.4332 (8) Å and a centroid offset of 1.946 Å.

From this analysis, it is evident that sulfonates, as usual has a penchant for the formation of bidentate motif and the intermolecular interactions involved in this structure paves way to the formation of two different hydrogen bonded arrays. Identification of such patterns will help in design and construction of preferred hydrogen bonding patterns on drug like molecules.

### Experimental

To obtain crystals of compound (I) suitable for X-ray study, pyrimethamine (31 mg; Shah Pharma Chemicals, India) was dissolved in hot n-propanol (20 ml) and 3-pyridine sulf77onic acid (20 mg; Merck) was dissolved in hot n-propanol (20 ml). The two solutions were mixed and warmed for 20 minutes over a water bath. The solution was allowed to evaporate slowly. After a few days, colourless crystals were obtained.

### Refinement

All the hydrogen atoms were positioned geometrically and were refined using riding model. The N—H and C—H bond lengths are 0.86 and 0.93 Å, respectively [*U*_iso_(H) = 1.2*U*_eq_(parent atom)].

### Crystal data

**Table:**

C_12_H_14_ClN_4_^+^·C_5_H_4_NO_3_S^−^	*Z* = 4
*M**_r_* = 407.88	*F*(000) = 848
Triclinic, *P*1	*D*_x_ = 1.398 Mg m^−^^3^
Hall symbol: -P 1	Mo *K*α radiation, λ = 0.71073 Å
*a* = 10.4525 (17) Å	Cell parameters from 11491 reflections
*b* = 12.200 (2) Å	θ = 1.8–31.3°
*c* = 16.539 (3) Å	µ = 0.33 mm^−^^1^
α = 81.130 (9)°	*T* = 296 K
β = 83.580 (9)°	Prism, colourless
γ = 68.649 (8)°	0.22 × 0.17 × 0.15 mm
*V* = 1937.3 (6) Å^3^	

### Data collection

**Table:**

Bruker SMART APEXII CCD area-detector diffractometer	7509 reflections with *I* > 2σ(*I*)
φ and ω scans	*R*_int_ = 0.038
Absorption correction: multi-scan (*SADABS*; Bruker, 2008)	θ_max_ = 31.3°, θ_min_ = 1.8°
*T*_min_ = 0.930, *T*_max_ = 0.952	*h* = −15→15
38297 measured reflections	*k* = −17→17
11491 independent reflections	*l* = −23→23

### Refinement

**Table:**

Refinement on *F*^2^	Primary atom site location: structure-invariant direct methods
Least-squares matrix: full	Secondary atom site location: difference Fourier map
*R*[*F*^2^ > 2σ(*F*^2^)] = 0.048	Hydrogen site location: inferred from neighbouring sites
*wR*(*F*^2^) = 0.142	H-atom parameters constrained
*S* = 1.04	*w* = 1/[σ^2^(*F*_o_^2^) + (0.0646*P*)^2^ + 0.3968*P*] where *P* = (*F*_o_^2^ + 2*F*_c_^2^)/3
11491 reflections	(Δ/σ)_max_ < 0.001
489 parameters	Δρ_max_ = 0.36 e Å^−^^3^
0 restraints	Δρ_min_ = −0.39 e Å^−^^3^

### Special details

**Table:**

Geometry. Bond distances, angles *etc*. have been calculated using the rounded fractional coordinates. All su's are estimated from the variances of the (full) variance-covariance matrix. The cell e.s.d.'s are taken into account in the estimation of distances, angles and torsion angles
Refinement. Refinement on *F*^2^ for ALL reflections except those flagged by the user for potential systematic errors. Weighted *R*-factors *wR* and all goodnesses of fit *S* are based on *F*^2^, conventional *R*-factors *R* are based on *F*, with *F* set to zero for negative *F*^2^. The observed criterion of *F*^2^ > σ(*F*^2^) is used only for calculating -*R*-factor-obs *etc*. and is not relevant to the choice of reflections for refinement. *R*-factors based on *F*^2^ are statistically about twice as large as those based on *F*, and *R*-factors based on ALL data will be even larger.

### Fractional atomic coordinates and isotropic or equivalent isotropic displacement parameters (Å^2^)

**Table:**

	*x*	*y*	*z*	*U*_iso_*/*U*_eq_	
Cl1A	0.05343 (7)	0.89280 (6)	0.09532 (3)	0.0782 (3)	
N1A	0.08451 (14)	0.63858 (13)	0.58329 (8)	0.0410 (4)	
N2A	0.23893 (15)	0.51659 (15)	0.67551 (9)	0.0482 (5)	
N3A	0.31757 (14)	0.55476 (13)	0.54244 (8)	0.0412 (4)	
N4A	0.39218 (16)	0.59433 (17)	0.41159 (9)	0.0579 (5)	
C2A	0.21517 (16)	0.56952 (15)	0.60000 (10)	0.0381 (5)	
C4A	0.28738 (17)	0.61007 (16)	0.46654 (10)	0.0404 (5)	
C5A	0.14942 (16)	0.68001 (15)	0.44467 (10)	0.0381 (5)	
C6A	0.04881 (16)	0.69257 (15)	0.50596 (10)	0.0384 (5)	
C7A	−0.10156 (18)	0.75762 (18)	0.49834 (12)	0.0497 (6)	
C8A	−0.1822 (2)	0.6740 (2)	0.51015 (15)	0.0714 (9)	
C9A	0.12152 (17)	0.73574 (15)	0.35859 (10)	0.0394 (5)	
C10A	0.0526 (2)	0.69537 (19)	0.31033 (12)	0.0566 (7)	
C11A	0.0286 (3)	0.7442 (2)	0.22982 (12)	0.0626 (8)	
C12A	0.0762 (2)	0.83365 (18)	0.19790 (11)	0.0491 (6)	
C13A	0.1449 (2)	0.87560 (19)	0.24377 (13)	0.0581 (7)	
C14A	0.1670 (2)	0.82680 (18)	0.32428 (12)	0.0529 (6)	
Cl1B	0.75619 (13)	0.10989 (8)	0.46283 (4)	0.1200 (4)	
N1B	0.58495 (16)	0.29629 (14)	−0.01927 (9)	0.0464 (5)	
N2B	0.63275 (18)	0.41465 (15)	−0.13246 (9)	0.0541 (6)	
N3B	0.69002 (16)	0.43241 (14)	−0.00694 (9)	0.0466 (5)	
N4B	0.7508 (2)	0.44828 (16)	0.11694 (10)	0.0606 (6)	
C2B	0.63649 (18)	0.38129 (16)	−0.05257 (11)	0.0432 (5)	
C4B	0.69761 (19)	0.39431 (17)	0.07335 (11)	0.0451 (6)	
C5B	0.65112 (19)	0.29990 (16)	0.11129 (11)	0.0445 (6)	
C6B	0.59195 (19)	0.25441 (17)	0.06234 (11)	0.0453 (6)	
C7B	0.5359 (2)	0.15697 (19)	0.08912 (13)	0.0589 (7)	
C8B	0.6301 (3)	0.0416 (2)	0.06264 (19)	0.0887 (11)	
C9B	0.6738 (2)	0.25302 (17)	0.19933 (11)	0.0473 (6)	
C10B	0.5798 (2)	0.3005 (2)	0.26134 (13)	0.0619 (7)	
C11B	0.6061 (3)	0.2556 (2)	0.34251 (14)	0.0724 (9)	
C12B	0.7232 (3)	0.1654 (2)	0.36127 (13)	0.0703 (9)	
C13B	0.8169 (4)	0.1170 (3)	0.30171 (18)	0.1075 (11)	
C14B	0.7925 (3)	0.1609 (3)	0.22042 (16)	0.0897 (10)	
S1A	0.12542 (4)	0.38315 (4)	0.22968 (3)	0.0454 (1)	
O1A	0.11857 (15)	0.28908 (13)	0.29387 (8)	0.0619 (5)	
O2A	0.00169 (14)	0.48731 (12)	0.23174 (9)	0.0592 (5)	
O3A	0.24940 (15)	0.40950 (17)	0.22690 (9)	0.0706 (6)	
N17A	0.1545 (2)	0.3676 (2)	−0.00909 (11)	0.0774 (8)	
C15A	0.12956 (18)	0.32917 (17)	0.13658 (11)	0.0440 (5)	
C16A	0.1441 (3)	0.3989 (2)	0.06510 (12)	0.0635 (8)	
C18A	0.1467 (3)	0.2638 (3)	−0.01309 (15)	0.0816 (11)	
C19A	0.1299 (4)	0.1884 (3)	0.05338 (17)	0.0943 (13)	
C20A	0.1217 (3)	0.2214 (2)	0.13088 (15)	0.0734 (9)	
S1B	0.56043 (5)	0.75098 (4)	0.20892 (2)	0.0410 (1)	
O1B	0.48714 (17)	0.81153 (12)	0.13616 (8)	0.0612 (5)	
O2B	0.50786 (13)	0.65999 (11)	0.25133 (7)	0.0459 (4)	
O3B	0.70717 (14)	0.70587 (14)	0.19638 (9)	0.0615 (5)	
N17B	0.5612 (3)	0.9012 (2)	0.40602 (16)	0.1179 (12)	
C15B	0.52136 (19)	0.85892 (16)	0.27683 (11)	0.0446 (6)	
C16B	0.5830 (3)	0.8278 (2)	0.34985 (16)	0.0970 (12)	
C18B	0.4734 (3)	1.0104 (2)	0.38887 (16)	0.0842 (10)	
C19B	0.4067 (2)	1.0492 (2)	0.31906 (15)	0.0657 (8)	
C20B	0.4310 (2)	0.97223 (18)	0.26126 (12)	0.0530 (6)	
H2A2	0.32110	0.47210	0.68780	0.0580*	
H1A	0.02160	0.64900	0.62260	0.0490*	
H2A1	0.17220	0.52660	0.71240	0.0580*	
H4A1	0.47390	0.55070	0.42540	0.0690*	
H4A2	0.37880	0.62770	0.36200	0.0690*	
H7A1	−0.11660	0.80330	0.44460	0.0600*	
H7A2	−0.13550	0.81270	0.53900	0.0600*	
H8A1	−0.15070	0.62080	0.46910	0.1070*	
H8A2	−0.27830	0.71920	0.50530	0.1070*	
H8A3	−0.16840	0.62920	0.56360	0.1070*	
H10A	0.02160	0.63400	0.33240	0.0680*	
H11A	−0.01880	0.71700	0.19800	0.0750*	
H13A	0.17640	0.93640	0.22100	0.0700*	
H14A	0.21310	0.85560	0.35590	0.0640*	
H1B	0.54690	0.26800	−0.05020	0.0560*	
H2B2	0.66470	0.46880	−0.15430	0.0650*	
H10B	0.49840	0.36280	0.24880	0.0740*	
H2B1	0.59830	0.38220	−0.16260	0.0650*	
H11B	0.54230	0.28800	0.38420	0.0870*	
H4B1	0.77810	0.50450	0.09330	0.0730*	
H4B2	0.75790	0.42700	0.16880	0.0730*	
H13B	0.89750	0.05440	0.31520	0.1290*	
H7B1	0.52190	0.14810	0.14840	0.0710*	
H14B	0.85740	0.12750	0.17950	0.1080*	
H7B2	0.44730	0.17830	0.06630	0.0710*	
H8B1	0.64770	0.05090	0.00420	0.1330*	
H8B2	0.58780	−0.01720	0.07780	0.1330*	
H8B3	0.71520	0.01680	0.08890	0.1330*	
H16A	0.14690	0.47320	0.06920	0.0760*	
H18A	0.15310	0.24020	−0.06470	0.0980*	
H19A	0.12390	0.11600	0.04700	0.1130*	
H20A	0.11130	0.17160	0.17760	0.0880*	
H16B	0.64400	0.75050	0.36100	0.1170*	
H18B	0.45670	1.06330	0.42710	0.1010*	
H19B	0.34510	1.12660	0.30990	0.0790*	
H20B	0.38650	0.99720	0.21250	0.0640*	

### Atomic displacement parameters (Å^2^)

**Table:**

	*U*^11^	*U*^22^	*U*^33^	*U*^12^	*U*^13^	*U*^23^
Cl1A	0.1046 (5)	0.0904 (5)	0.0411 (3)	−0.0428 (4)	−0.0217 (3)	0.0212 (3)
N1A	0.0334 (7)	0.0503 (8)	0.0321 (7)	−0.0093 (6)	0.0018 (5)	−0.0007 (6)
N2A	0.0389 (7)	0.0622 (10)	0.0339 (7)	−0.0116 (7)	−0.0026 (6)	0.0072 (7)
N3A	0.0345 (7)	0.0493 (8)	0.0329 (7)	−0.0096 (6)	−0.0016 (5)	0.0024 (6)
N4A	0.0390 (8)	0.0808 (12)	0.0349 (8)	−0.0068 (8)	0.0020 (6)	0.0096 (8)
C2A	0.0368 (8)	0.0421 (9)	0.0325 (8)	−0.0125 (7)	−0.0024 (6)	0.0005 (6)
C4A	0.0388 (8)	0.0454 (9)	0.0317 (8)	−0.0112 (7)	−0.0004 (6)	0.0005 (7)
C5A	0.0387 (8)	0.0395 (9)	0.0326 (8)	−0.0112 (7)	−0.0036 (6)	0.0002 (6)
C6A	0.0372 (8)	0.0390 (9)	0.0349 (8)	−0.0097 (7)	−0.0042 (6)	−0.0001 (7)
C7A	0.0383 (9)	0.0561 (11)	0.0417 (9)	−0.0043 (8)	−0.0042 (7)	0.0028 (8)
C8A	0.0489 (12)	0.0947 (18)	0.0718 (15)	−0.0291 (12)	−0.0169 (10)	0.0049 (13)
C9A	0.0387 (8)	0.0407 (9)	0.0328 (8)	−0.0088 (7)	−0.0036 (6)	0.0010 (7)
C10A	0.0869 (15)	0.0535 (12)	0.0402 (10)	−0.0398 (11)	−0.0100 (9)	0.0041 (8)
C11A	0.0951 (16)	0.0649 (13)	0.0407 (10)	−0.0424 (13)	−0.0203 (10)	0.0027 (9)
C12A	0.0568 (11)	0.0518 (11)	0.0333 (9)	−0.0157 (9)	−0.0069 (7)	0.0051 (7)
C13A	0.0658 (12)	0.0607 (13)	0.0526 (11)	−0.0347 (11)	−0.0144 (9)	0.0173 (9)
C14A	0.0589 (11)	0.0599 (12)	0.0469 (10)	−0.0312 (10)	−0.0171 (8)	0.0086 (9)
Cl1B	0.2271 (11)	0.0951 (6)	0.0505 (4)	−0.0703 (7)	−0.0469 (5)	0.0151 (3)
N1B	0.0584 (9)	0.0509 (9)	0.0392 (8)	−0.0287 (8)	−0.0065 (6)	−0.0069 (6)
N2B	0.0722 (11)	0.0635 (11)	0.0385 (8)	−0.0382 (9)	−0.0082 (7)	−0.0021 (7)
N3B	0.0583 (9)	0.0490 (9)	0.0398 (8)	−0.0270 (8)	−0.0044 (6)	−0.0055 (6)
N4B	0.0897 (13)	0.0692 (11)	0.0412 (9)	−0.0486 (10)	−0.0092 (8)	−0.0057 (8)
C2B	0.0480 (9)	0.0447 (10)	0.0385 (9)	−0.0180 (8)	−0.0028 (7)	−0.0058 (7)
C4B	0.0516 (10)	0.0474 (10)	0.0403 (9)	−0.0213 (8)	−0.0034 (7)	−0.0074 (7)
C5B	0.0508 (10)	0.0473 (10)	0.0384 (9)	−0.0213 (8)	−0.0009 (7)	−0.0059 (7)
C6B	0.0492 (10)	0.0474 (10)	0.0419 (9)	−0.0207 (8)	−0.0008 (7)	−0.0057 (7)
C7B	0.0745 (14)	0.0640 (13)	0.0510 (11)	−0.0421 (12)	−0.0030 (10)	−0.0011 (9)
C8B	0.112 (2)	0.0606 (16)	0.099 (2)	−0.0420 (16)	0.0015 (17)	−0.0028 (14)
C9B	0.0571 (11)	0.0493 (10)	0.0397 (9)	−0.0239 (9)	−0.0046 (8)	−0.0036 (8)
C10B	0.0640 (13)	0.0708 (14)	0.0458 (11)	−0.0188 (11)	−0.0021 (9)	−0.0054 (10)
C11B	0.0957 (18)	0.0831 (17)	0.0428 (11)	−0.0387 (15)	0.0078 (11)	−0.0117 (11)
C12B	0.114 (2)	0.0620 (14)	0.0438 (11)	−0.0413 (15)	−0.0214 (12)	0.0047 (10)
C13B	0.110 (2)	0.106 (2)	0.0650 (18)	0.0125 (19)	−0.0283 (16)	0.0023 (16)
C14B	0.0827 (18)	0.096 (2)	0.0533 (14)	0.0109 (15)	−0.0057 (12)	−0.0051 (13)
S1A	0.0431 (2)	0.0546 (3)	0.0372 (2)	−0.0179 (2)	0.0054 (2)	−0.0056 (2)
O1A	0.0667 (9)	0.0621 (9)	0.0411 (7)	−0.0117 (7)	0.0101 (6)	0.0033 (6)
O2A	0.0578 (8)	0.0481 (8)	0.0658 (9)	−0.0153 (7)	0.0151 (7)	−0.0121 (7)
O3A	0.0586 (9)	0.1133 (14)	0.0532 (9)	−0.0460 (9)	−0.0021 (7)	−0.0110 (8)
N17A	0.1227 (18)	0.0894 (15)	0.0420 (10)	−0.0650 (14)	0.0033 (10)	−0.0098 (9)
C15A	0.0429 (9)	0.0496 (10)	0.0410 (9)	−0.0199 (8)	0.0066 (7)	−0.0074 (7)
C16A	0.0936 (16)	0.0665 (14)	0.0436 (11)	−0.0458 (13)	0.0013 (10)	−0.0062 (9)
C18A	0.115 (2)	0.102 (2)	0.0560 (14)	−0.0695 (18)	0.0113 (13)	−0.0265 (13)
C19A	0.153 (3)	0.091 (2)	0.0725 (17)	−0.082 (2)	0.0202 (17)	−0.0313 (15)
C20A	0.109 (2)	0.0648 (14)	0.0579 (13)	−0.0491 (14)	0.0126 (12)	−0.0085 (11)
S1B	0.0532 (3)	0.0443 (2)	0.0281 (2)	−0.0208 (2)	−0.0059 (2)	−0.0003 (2)
O1B	0.1014 (11)	0.0536 (8)	0.0365 (7)	−0.0350 (8)	−0.0274 (7)	0.0067 (6)
O2B	0.0559 (7)	0.0476 (7)	0.0366 (6)	−0.0233 (6)	−0.0040 (5)	0.0009 (5)
O3B	0.0549 (8)	0.0749 (10)	0.0558 (8)	−0.0262 (8)	0.0110 (6)	−0.0135 (7)
N17B	0.177 (3)	0.0741 (15)	0.0801 (16)	0.0096 (16)	−0.0707 (17)	−0.0327 (13)
C15B	0.0531 (10)	0.0440 (10)	0.0369 (9)	−0.0155 (8)	−0.0119 (7)	−0.0030 (7)
C16B	0.138 (3)	0.0592 (15)	0.0711 (16)	0.0149 (15)	−0.0631 (17)	−0.0243 (12)
C18B	0.123 (2)	0.0581 (15)	0.0658 (16)	−0.0144 (15)	−0.0211 (15)	−0.0235 (12)
C19B	0.0717 (14)	0.0449 (11)	0.0721 (15)	−0.0082 (10)	−0.0084 (11)	−0.0109 (10)
C20B	0.0568 (11)	0.0505 (11)	0.0500 (11)	−0.0169 (9)	−0.0138 (9)	0.0014 (9)

### Geometric parameters (Å, °)

**Table:**

Cl1A—C12A	1.7445 (19)	C13A—C14A	1.380 (3)
Cl1B—C12B	1.734 (2)	C7A—H7A1	0.9700
S1A—O2A	1.4475 (15)	C7A—H7A2	0.9700
S1A—O3A	1.4389 (18)	C8A—H8A3	0.9600
S1A—C15A	1.7560 (19)	C8A—H8A2	0.9600
S1A—O1A	1.4548 (15)	C8A—H8A1	0.9600
S1B—O1B	1.4494 (15)	C10A—H10A	0.9300
S1B—O2B	1.4643 (14)	C11A—H11A	0.9300
S1B—C15B	1.7676 (19)	C13A—H13A	0.9300
S1B—O3B	1.4310 (17)	C14A—H14A	0.9300
N1A—C6A	1.370 (2)	C4B—C5B	1.440 (3)
N1A—C2A	1.351 (2)	C5B—C9B	1.492 (3)
N2A—C2A	1.320 (2)	C5B—C6B	1.359 (3)
N3A—C4A	1.343 (2)	C6B—C7B	1.496 (3)
N3A—C2A	1.330 (2)	C7B—C8B	1.492 (3)
N4A—C4A	1.319 (2)	C9B—C10B	1.377 (3)
N1A—H1A	0.8600	C9B—C14B	1.373 (4)
N2A—H2A1	0.8600	C10B—C11B	1.387 (3)
N2A—H2A2	0.8600	C11B—C12B	1.343 (4)
N4A—H4A1	0.8600	C12B—C13B	1.349 (4)
N4A—H4A2	0.8600	C13B—C14B	1.384 (4)
N1B—C6B	1.368 (2)	C7B—H7B2	0.9700
N1B—C2B	1.354 (3)	C7B—H7B1	0.9700
N2B—C2B	1.322 (2)	C8B—H8B1	0.9600
N3B—C4B	1.339 (2)	C8B—H8B3	0.9600
N3B—C2B	1.329 (3)	C8B—H8B2	0.9600
N4B—C4B	1.329 (3)	C10B—H10B	0.9300
N1B—H1B	0.8600	C11B—H11B	0.9300
N2B—H2B1	0.8600	C13B—H13B	0.9300
N2B—H2B2	0.8600	C14B—H14B	0.9300
N4B—H4B2	0.8600	C15A—C16A	1.374 (3)
N4B—H4B1	0.8600	C15A—C20A	1.365 (3)
N17A—C16A	1.322 (3)	C18A—C19A	1.360 (4)
N17A—C18A	1.310 (4)	C19A—C20A	1.386 (4)
N17B—C18B	1.323 (3)	C16A—H16A	0.9300
N17B—C16B	1.333 (4)	C18A—H18A	0.9300
C4A—C5A	1.437 (3)	C19A—H19A	0.9300
C5A—C9A	1.491 (2)	C20A—H20A	0.9300
C5A—C6A	1.360 (2)	C15B—C16B	1.368 (3)
C6A—C7A	1.490 (3)	C15B—C20B	1.365 (3)
C7A—C8A	1.522 (3)	C18B—C19B	1.347 (4)
C9A—C14A	1.383 (3)	C19B—C20B	1.382 (3)
C9A—C10A	1.381 (3)	C16B—H16B	0.9300
C10A—C11A	1.383 (3)	C18B—H18B	0.9300
C11A—C12A	1.370 (3)	C19B—H19B	0.9300
C12A—C13A	1.365 (3)	C20B—H20B	0.9300
			
Cl1B···C4A^i^	3.636 (2)	N3B···C16A^iv^	3.128 (3)
Cl1B···C5A^i^	3.643 (2)	N4A···O2B	2.909 (2)
Cl1A···H19A^ii^	3.0400	N4A···C14A	3.210 (3)
S1A···H2A1^iii^	3.0000	N4A···N3A^i^	3.027 (2)
S1A···H1A^iii^	2.7900	N4B···N17A^iv^	3.033 (3)
S1B···H1B^iv^	3.0400	N17A···N4B^iv^	3.033 (3)
S1B···H2A2^i^	2.9100	N1A···H8A3	2.7400
S1B···H2B1^iv^	2.9300	N1B···H8B1	2.8000
O1A···C19B^v^	3.361 (3)	N2A···H4B2^i^	2.7700
O1A···N1A^iii^	2.764 (2)	N3A···H4A1^i^	2.1700
O1A···C7A^iii^	3.391 (2)	N3B···H16A^iv^	2.4400
O1B···N1B^iv^	2.805 (2)	N17A···H4B1^iv^	2.1900
O2A···N2A^iii^	2.808 (2)	N17B···H18B^vii^	2.8300
O2A···C11A	3.242 (3)	N17B···H8A2^vi^	2.6900
O2A···C10A	3.251 (3)	C2A···C11B^i^	3.588 (3)
O2B···N2B^iv^	3.005 (2)	C2B···C4B^iv^	3.581 (3)
O2B···N2A^i^	2.978 (2)	C4A···Cl1B^i^	3.636 (2)
O2B···N4A	2.909 (2)	C4B···C2B^iv^	3.581 (3)
O3A···C10B	3.299 (3)	C5A···Cl1B^i^	3.643 (2)
O3A···N2B^iv^	3.003 (3)	C7A···O1A^iii^	3.391 (2)
O3B···N2A^i^	3.091 (2)	C7A···C10A	3.400 (3)
O3B···C18A^iv^	3.270 (3)	C10A···C7A	3.400 (3)
O1A···H1A^iii^	1.9200	C10A···O2A	3.251 (3)
O1A···H20A	2.5900	C10B···O3A	3.299 (3)
O1A···H19B^v^	2.4800	C11A···O2A	3.242 (3)
O1A···H8A3^iii^	2.8600	C11B···C2A^i^	3.588 (3)
O1A···H7A2^iii^	2.8500	C14A···N4A	3.210 (3)
O1B···H8B1^iv^	2.8300	C16A···N3B^iv^	3.128 (3)
O1B···H20B	2.5800	C18A···O3B^iv^	3.270 (3)
O1B···H1B^iv^	1.9700	C19B···O1A^ii^	3.361 (3)
O1B···H8B2^ii^	2.6800	C8A···H1A	2.8900
O1B···H2B1^iv^	2.7800	C8B···H1B	2.9800
O2A···H10A	2.7000	C9A···H7A1	2.6400
O2A···H11A	2.7100	C9A···H4A2	2.5300
O2A···H2A1^iii^	2.0000	C9B···H4B2	2.5400
O2A···H1A^iii^	2.7500	C9B···H7B1	2.6400
O2B···H2A2^i^	2.1500	C10A···H7A1	2.8500
O2B···H2B1^iv^	2.1600	C10B···H4B2	2.9900
O2B···H4A2	2.2200	C13A···H13B^viii^	2.9700
O3A···H10B	2.5100	C14A···H4A2	2.6700
O3A···H16A	2.8200	C16A···H4B1^iv^	2.8600
O3A···H2B2^iv^	2.1500	C16B···H8A2^vi^	2.9800
O3B···H2A1^i^	2.9000	C20B···H7B1^ii^	2.9700
O3B···H2A2^i^	2.7400	H1A···H8A3	2.4100
O3B···H16B	2.8200	H1A···H2A1	2.2500
O3B···H11A^vi^	2.9200	H1A···H7A2	2.4200
O3B···H18A^iv^	2.6200	H1B···H2B1	2.2800
N1A···O1A^iii^	2.764 (2)	H1B···H7B2	2.3900
N1B···O1B^iv^	2.805 (2)	H1B···H8B1	2.5300
N2A···O2A^iii^	2.808 (2)	H2A1···H4B2^i^	2.3800
N2A···O3B^i^	3.091 (2)	H2A1···H1A	2.2500
N2A···O2B^i^	2.978 (2)	H7A2···H1A	2.4200
N2B···O3A^iv^	3.003 (3)	H8A2···H16B^ix^	2.5400
N2B···O2B^iv^	3.005 (2)	H2B2···H10B^iv^	2.5700
N3A···N4A^i^	3.027 (2)	H13A···H20B	2.5500
			
O3A—S1A—C15A	105.74 (10)	C13A—C14A—H14A	120.00
O1A—S1A—O3A	113.82 (10)	C9A—C14A—H14A	120.00
O1A—S1A—C15A	105.92 (9)	N1B—C2B—N2B	119.11 (18)
O1A—S1A—O2A	111.57 (9)	N2B—C2B—N3B	119.20 (18)
O2A—S1A—C15A	105.93 (9)	N1B—C2B—N3B	121.68 (16)
O2A—S1A—O3A	113.08 (10)	N3B—C4B—N4B	116.46 (18)
O2B—S1B—O3B	111.80 (9)	N3B—C4B—C5B	122.36 (18)
O1B—S1B—C15B	105.88 (9)	N4B—C4B—C5B	121.18 (17)
O1B—S1B—O3B	114.99 (9)	C6B—C5B—C9B	122.95 (18)
O2B—S1B—C15B	105.67 (8)	C4B—C5B—C9B	120.27 (17)
O3B—S1B—C15B	106.12 (10)	C4B—C5B—C6B	116.73 (17)
O1B—S1B—O2B	111.61 (9)	C5B—C6B—C7B	125.56 (17)
C2A—N1A—C6A	122.28 (15)	N1B—C6B—C7B	115.24 (17)
C2A—N3A—C4A	118.01 (16)	N1B—C6B—C5B	119.17 (18)
C6A—N1A—H1A	119.00	C6B—C7B—C8B	112.0 (2)
C2A—N1A—H1A	119.00	C5B—C9B—C10B	122.07 (19)
H2A2—N2A—H2A1	120.00	C10B—C9B—C14B	118.13 (19)
C2A—N2A—H2A1	120.00	C5B—C9B—C14B	119.80 (19)
C2A—N2A—H2A2	120.00	C9B—C10B—C11B	120.2 (2)
C4A—N4A—H4A2	120.00	C10B—C11B—C12B	120.3 (2)
H4A1—N4A—H4A2	120.00	C11B—C12B—C13B	120.7 (2)
C4A—N4A—H4A1	120.00	Cl1B—C12B—C11B	120.23 (19)
C2B—N1B—C6B	121.60 (17)	Cl1B—C12B—C13B	119.1 (2)
C2B—N3B—C4B	118.31 (17)	C12B—C13B—C14B	119.7 (3)
C6B—N1B—H1B	119.00	C9B—C14B—C13B	120.9 (3)
C2B—N1B—H1B	119.00	C8B—C7B—H7B2	109.00
C2B—N2B—H2B1	120.00	C8B—C7B—H7B1	109.00
H2B2—N2B—H2B1	120.00	C6B—C7B—H7B2	109.00
C2B—N2B—H2B2	120.00	C6B—C7B—H7B1	109.00
H4B1—N4B—H4B2	120.00	H7B1—C7B—H7B2	108.00
C4B—N4B—H4B1	120.00	H8B1—C8B—H8B3	110.00
C4B—N4B—H4B2	120.00	C7B—C8B—H8B1	109.00
C16A—N17A—C18A	116.4 (2)	C7B—C8B—H8B2	109.00
C16B—N17B—C18B	116.7 (3)	C7B—C8B—H8B3	109.00
N1A—C2A—N3A	121.39 (15)	H8B1—C8B—H8B2	109.00
N1A—C2A—N2A	118.09 (16)	H8B2—C8B—H8B3	109.00
N2A—C2A—N3A	120.52 (16)	C9B—C10B—H10B	120.00
N3A—C4A—N4A	116.04 (17)	C11B—C10B—H10B	120.00
N4A—C4A—C5A	121.14 (16)	C12B—C11B—H11B	120.00
N3A—C4A—C5A	122.80 (16)	C10B—C11B—H11B	120.00
C4A—C5A—C9A	120.40 (15)	C12B—C13B—H13B	120.00
C4A—C5A—C6A	116.69 (15)	C14B—C13B—H13B	120.00
C6A—C5A—C9A	122.91 (16)	C13B—C14B—H14B	120.00
N1A—C6A—C7A	114.49 (15)	C9B—C14B—H14B	120.00
C5A—C6A—C7A	126.76 (16)	C16A—C15A—C20A	118.02 (19)
N1A—C6A—C5A	118.72 (16)	S1A—C15A—C16A	117.92 (16)
C6A—C7A—C8A	112.06 (17)	S1A—C15A—C20A	124.05 (16)
C5A—C9A—C10A	120.68 (16)	N17A—C16A—C15A	124.5 (2)
C10A—C9A—C14A	118.25 (17)	N17A—C18A—C19A	124.2 (3)
C5A—C9A—C14A	121.05 (17)	C18A—C19A—C20A	118.8 (3)
C9A—C10A—C11A	121.5 (2)	C15A—C20A—C19A	118.1 (2)
C10A—C11A—C12A	118.5 (2)	N17A—C16A—H16A	118.00
Cl1A—C12A—C11A	119.49 (17)	C15A—C16A—H16A	118.00
Cl1A—C12A—C13A	118.89 (16)	N17A—C18A—H18A	118.00
C11A—C12A—C13A	121.59 (19)	C19A—C18A—H18A	118.00
C12A—C13A—C14A	119.3 (2)	C18A—C19A—H19A	121.00
C9A—C14A—C13A	120.91 (19)	C20A—C19A—H19A	121.00
C8A—C7A—H7A2	109.00	C15A—C20A—H20A	121.00
C6A—C7A—H7A2	109.00	C19A—C20A—H20A	121.00
C8A—C7A—H7A1	109.00	S1B—C15B—C16B	118.77 (16)
C6A—C7A—H7A1	109.00	S1B—C15B—C20B	123.70 (15)
H7A1—C7A—H7A2	108.00	C16B—C15B—C20B	117.52 (18)
C7A—C8A—H8A3	109.00	N17B—C16B—C15B	124.2 (2)
H8A1—C8A—H8A3	109.00	N17B—C18B—C19B	123.5 (2)
C7A—C8A—H8A2	109.00	C18B—C19B—C20B	119.0 (2)
H8A1—C8A—H8A2	109.00	C15B—C20B—C19B	118.99 (19)
C7A—C8A—H8A1	109.00	N17B—C16B—H16B	118.00
H8A2—C8A—H8A3	109.00	C15B—C16B—H16B	118.00
C9A—C10A—H10A	119.00	N17B—C18B—H18B	118.00
C11A—C10A—H10A	119.00	C19B—C18B—H18B	118.00
C10A—C11A—H11A	121.00	C18B—C19B—H19B	121.00
C12A—C11A—H11A	121.00	C20B—C19B—H19B	120.00
C12A—C13A—H13A	120.00	C15B—C20B—H20B	120.00
C14A—C13A—H13A	120.00	C19B—C20B—H20B	121.00
			
O2A—S1A—C15A—C16A	65.0 (2)	C5A—C9A—C10A—C11A	−178.5 (2)
O1A—S1A—C15A—C16A	−176.4 (2)	C10A—C9A—C14A—C13A	−0.4 (3)
O1A—S1A—C15A—C20A	2.4 (2)	C14A—C9A—C10A—C11A	−0.2 (3)
O3A—S1A—C15A—C20A	123.5 (2)	C5A—C9A—C14A—C13A	177.88 (19)
O2A—S1A—C15A—C20A	−116.2 (2)	C9A—C10A—C11A—C12A	0.7 (4)
O3A—S1A—C15A—C16A	−55.3 (2)	C10A—C11A—C12A—C13A	−0.6 (4)
O1B—S1B—C15B—C16B	−176.6 (2)	C10A—C11A—C12A—Cl1A	177.44 (18)
O2B—S1B—C15B—C16B	64.8 (2)	Cl1A—C12A—C13A—C14A	−178.06 (17)
O2B—S1B—C15B—C20B	−114.20 (19)	C11A—C12A—C13A—C14A	0.0 (3)
O1B—S1B—C15B—C20B	4.3 (2)	C12A—C13A—C14A—C9A	0.5 (3)
O3B—S1B—C15B—C20B	126.96 (19)	N3B—C4B—C5B—C6B	−3.3 (3)
O3B—S1B—C15B—C16B	−54.0 (2)	N4B—C4B—C5B—C9B	−5.7 (3)
C6A—N1A—C2A—N2A	−176.88 (16)	N4B—C4B—C5B—C6B	176.8 (2)
C2A—N1A—C6A—C7A	175.61 (16)	N3B—C4B—C5B—C9B	174.24 (19)
C2A—N1A—C6A—C5A	−2.7 (3)	C9B—C5B—C6B—N1B	−174.81 (18)
C6A—N1A—C2A—N3A	3.1 (3)	C4B—C5B—C6B—N1B	2.6 (3)
C2A—N3A—C4A—N4A	179.13 (17)	C4B—C5B—C6B—C7B	−179.57 (19)
C2A—N3A—C4A—C5A	−2.5 (3)	C9B—C5B—C6B—C7B	3.0 (3)
C4A—N3A—C2A—N1A	−0.4 (3)	C6B—C5B—C9B—C10B	−93.4 (3)
C4A—N3A—C2A—N2A	179.56 (17)	C4B—C5B—C9B—C10B	89.3 (3)
C2B—N1B—C6B—C7B	−177.49 (18)	C4B—C5B—C9B—C14B	−89.7 (3)
C6B—N1B—C2B—N2B	176.88 (18)	C6B—C5B—C9B—C14B	87.7 (3)
C6B—N1B—C2B—N3B	−3.5 (3)	C5B—C6B—C7B—C8B	−103.5 (3)
C2B—N1B—C6B—C5B	0.5 (3)	N1B—C6B—C7B—C8B	74.4 (3)
C4B—N3B—C2B—N2B	−177.53 (19)	C10B—C9B—C14B—C13B	0.0 (4)
C2B—N3B—C4B—N4B	−179.51 (19)	C5B—C9B—C14B—C13B	179.0 (3)
C2B—N3B—C4B—C5B	0.5 (3)	C5B—C9B—C10B—C11B	−178.8 (2)
C4B—N3B—C2B—N1B	2.8 (3)	C14B—C9B—C10B—C11B	0.1 (4)
C16A—N17A—C18A—C19A	−0.5 (5)	C9B—C10B—C11B—C12B	−0.1 (4)
C18A—N17A—C16A—C15A	1.8 (4)	C10B—C11B—C12B—Cl1B	179.7 (2)
C16B—N17B—C18B—C19B	0.2 (5)	C10B—C11B—C12B—C13B	−0.1 (5)
C18B—N17B—C16B—C15B	0.4 (5)	C11B—C12B—C13B—C14B	0.2 (5)
N3A—C4A—C5A—C6A	2.8 (3)	Cl1B—C12B—C13B—C14B	−179.5 (3)
N3A—C4A—C5A—C9A	−177.73 (16)	C12B—C13B—C14B—C9B	−0.2 (5)
N4A—C4A—C5A—C9A	0.6 (3)	C20A—C15A—C16A—N17A	−1.8 (4)
N4A—C4A—C5A—C6A	−178.94 (18)	S1A—C15A—C20A—C19A	−178.3 (3)
C4A—C5A—C6A—C7A	−178.24 (17)	S1A—C15A—C16A—N17A	177.1 (2)
C4A—C5A—C9A—C10A	110.3 (2)	C16A—C15A—C20A—C19A	0.5 (4)
C9A—C5A—C6A—N1A	−179.61 (16)	N17A—C18A—C19A—C20A	−0.7 (6)
C4A—C5A—C6A—N1A	−0.1 (2)	C18A—C19A—C20A—C15A	0.7 (5)
C9A—C5A—C6A—C7A	2.3 (3)	S1B—C15B—C16B—N17B	−179.7 (2)
C6A—C5A—C9A—C10A	−70.2 (2)	C20B—C15B—C16B—N17B	−0.6 (4)
C6A—C5A—C9A—C14A	111.5 (2)	S1B—C15B—C20B—C19B	179.24 (17)
C4A—C5A—C9A—C14A	−68.0 (2)	C16B—C15B—C20B—C19B	0.2 (3)
N1A—C6A—C7A—C8A	−67.9 (2)	N17B—C18B—C19B—C20B	−0.6 (4)
C5A—C6A—C7A—C8A	110.3 (2)	C18B—C19B—C20B—C15B	0.4 (3)

Symmetry codes: (i) −*x*+1, −*y*+1, −*z*+1; (ii) *x*, *y*+1, *z*; (iii) −*x*, −*y*+1, −*z*+1; (iv) −*x*+1, −*y*+1, −*z*; (v) *x*, *y*−1, *z*; (vi) *x*+1, *y*, *z*; (vii) −*x*+1, −*y*+2, −*z*+1; (viii) *x*−1, *y*+1, *z*; (ix) *x*−1, *y*, *z*.

### Hydrogen-bond geometry (Å, °)

**Table:**

*D*—H···*A*	*D*—H	H···*A*	*D*···*A*	*D*—H···*A*
N2A—H2A2···O2B^i^	0.86	2.15	2.978 (2)	162
N1A—H1A···O1A^iii^	0.86	1.92	2.764 (2)	166
N1B—H1B···O1B^iv^	0.86	1.97	2.805 (2)	164
N2A—H2A1···O2A^iii^	0.86	2.00	2.808 (2)	157
N4A—H4A1···N3A^i^	0.86	2.17	3.027 (2)	178
N4A—H4A2···O2B	0.86	2.22	2.909 (2)	137
N2B—H2B2···O3A^iv^	0.86	2.15	3.003 (3)	171
N2B—H2B1···O2B^iv^	0.86	2.16	3.005 (2)	167
N4B—H4B1···N17A^iv^	0.86	2.19	3.033 (3)	168
C10B—H10B···O3A	0.93	2.51	3.299 (3)	143
C16A—H16A···N3B^iv^	0.93	2.44	3.128 (3)	131
C19B—H19B···O1A^ii^	0.93	2.48	3.361 (3)	157

Symmetry codes: (i) −*x*+1, −*y*+1, −*z*+1; (iii) −*x*, −*y*+1, −*z*+1; (iv) −*x*+1, −*y*+1, −*z*; (ii) *x*, *y*+1, *z*.
